# *MSTN* and *TCF12* as Candidate Immunometabolic Signatures in Glioma-Associated Foam Cells: Insights from Integrated Multi-Omics Analysis

**DOI:** 10.3390/cimb48030289

**Published:** 2026-03-09

**Authors:** Xu Liu, Zhuo Song, Zhijia Sun, Chen Liu, Xiaoli Kang, Huilian Qiao, Xinzhuo Tu, Teng Li, Zhiguang Fu, Yingjie Wang

**Affiliations:** 1 Department of Radiotherapy Oncology, Air Force Medical Center, PLA, Air Force Medical University, Beijing 100142, China; xuxu_lh@163.com (X.L.); drzhuosong2013@126.com (Z.S.); sunzhijia301@163.com (Z.S.); liuchen_af@fmmu.edu.cn (C.L.); kangxioali-001@163.com (X.K.); 2 Department of Pathology, Air Force Medical Center, PLA, Air Force Medical University, Beijing 100142, China; qiaohi330@126.com (H.Q.); pathologytu@163.com (X.T.); jacksonlt@163.com (T.L.)

**Keywords:** tumor-associated foam cell, *MSTN*, *TCF12*, tumor microenvironment, immunometabolic regulation, glioma

## Abstract

The glioma tumor microenvironment (TME) exhibits profound heterogeneity that drives tumor progression and therapy resistance. By integrating single-cell RNA sequencing (eleven samples) and spatial transcriptomics (two samples), the cellular components of the glioma microenvironment were deconvoluted, revealing tumor-associated foam cells (TAFCs) as the most abundant and centrally connected subtype. The high expression of two prognostic candidate genes, growth differentiation factor 8 (GDF-8, also known as myostatin, *MSTN*) and transcription factor 12 (*TCF12*), in TAFCs was found to be correlated with poor overall survival. These two genes were associated with M2 macrophage infiltration, altered cholesterol homeostasis, and immunosuppressive signaling. Regulatory network and pathway analyses, based on computational motif enrichment and co-expression analysis, linked them to ribosome, Notch signaling, DNA repair, and cell-cycle pathways. Pseudotime trajectories revealed dynamic expression during differentiation. Additionally, drug sensitivity prediction analysis demonstrated that *MSTN* expression was significantly associated with sensitivity to paclitaxel and VE-822, while *TCF12* expression showed potential associations with sensitivity to cytarabine, olaparib, Wee1 inhibitor, paclitaxel, and VE-822. Logistic regression analysis combining clinical parameters with *MSTN* and *TCF12* expression effectively achieved risk stratification for glioma, with higher composite scores predicting worse 2- and 3-year survival outcomes. Calibration curves demonstrated high consistency between nomogram-predicted overall survival probabilities and actual observed outcomes. Immunofluorescence confirmed upregulated expression of *MSTN* and *TCF12* in glioma tissues and their co-localization with macrophages. In conclusion, this study identified TAFCs as the central cells in the glioma microenvironment, with their signature genes *MSTN* and *TCF12* representing candidate immunometabolic signatures associated with macrophage-mediated immunosuppression and metabolic reprogramming in glioma, suggesting their potential as biomarkers for patient stratification and as targets for immunometabolic therapies.

## 1. Introduction

Glioma represents the most devastating primary malignant tumor of the central nervous system, accounting for over 80% of all malignant brain tumors and posing a formidable clinical management challenge [1,2]. The molecular classification of glioma, as defined by the 2021 WHO diagnostic framework and prognostic stratification, incorporates markers such as IDH mutation status and 1p/19q co-deletion. Among gliomas, glioblastoma is the most aggressive subtype [3]. Despite the adoption of a standard therapeutic regimen based on maximal safe surgical resection followed by radiotherapy and temozolomide chemotherapy, the prognosis for high-grade glioma patients remains dismal, characterized by limited median survival and an exceedingly high recurrence rate [4]. Consequently, the etiology of this therapeutic challenge is increasingly attributed to the profound heterogeneity of the tumor and its intricate tumor microenvironment (TME). Thus, studying the pivotal cellular components within the TME to identify effective therapeutic targets and biomarkers is a central objective in this field [5,6,7]. A deeper mechanistic understanding of glioma biology, particularly within the context of its TME, is therefore regarded as fundamental for enabling early diagnosis and paving the way for personalized therapeutic strategies [5,8,9].

In the glioma TME, tumor-associated macrophages (TAMs) constitute the most abundant population of infiltrating immune cells, accounting for 50% of the tumor mass [9,10,11,12]. These cells play an indispensable yet complex and multifaceted role in tumor progression, traditionally conceptualized through the simplified M1/M2 polarization paradigm, wherein the anti-tumorigenic M1 phenotype contrasts with the pro-tumorigenic M2 phenotype [13,14]. M2-type TAMs serve as central architects of an immunosuppressive TME, primarily through the secretion of immunosuppressive cytokines (e.g., IL-10, TGF-β), promotion of angiogenesis, and facilitation of tissue remodeling [15,16,17]. However, this binary classification is far from sufficient to capture the functional complexity of TAMs. The rapid advancement of single-cell transcriptomics has fundamentally reshaped our understanding by unveiling remarkable functional diversity and phenotypic plasticity within this population [18,19,20]. Recent single-cell RNA sequencing studies have confirmed multiple distinct functional subpopulations beyond the M1/M2 framework, including interferon-stimulated, hypoxia-induced, and proliferative subtypes [18,21]. These subpopulations exhibit significant differences in spatial distribution, metabolic characteristics, and pro-tumoral functions, suggesting that effective TAM-targeted therapies require more precise, subpopulation-specific strategies rather than broad macrophage depletion or generic polarization modulation. Nevertheless, the research foundation supporting TAM diversity and function in glioma remains insufficient.

In this study, the metabolic heterogeneity and spatial architecture of the glioma tumor microenvironment were systematically dissected using an integrated multi-omics strategy, revealing tumor-associated foam cells (TAFCs) as the most abundant cellular subpopulation occupying a central position within the intercellular communication network. Through in-depth mining of TAFC-specific gene signatures, two core regulatory factors were identified: growth differentiation factor 8 (GDF-8, also known as myostatin, *MSTN*) and transcription factor 12 (*TCF12*). Elevated expression of these genes was associated with poor prognosis in glioma and they were found to be involved in multiple metabolic pathways and the regulation of the immune microenvironment. Immunofluorescence validation using independent clinical specimens confirmed that *MSTN* and *TCF12* were upregulated in glioma tissues and co-localized with macrophages. Collectively, this study provides a comprehensive characterization of the potential roles and underlying mechanisms of TAFCs and their key regulatory factors within the glioma immune microenvironment, thereby identifying candidate biomarkers for predicting therapeutic response and improving prognosis in glioma patients.

## 2. Materials and Methods

All the parameter settings and complete version information for all packages are listed in Table S1.

### 2.1. Datasets and Acquisition

The scRNA-seq data (GSE237673) from 11 glioma samples [22] and ST data (GSE270355) from 2 glioma cases [23] were downloaded from the GEO database. Bulk RNA-seq data, including data from 5 healthy brain tissue and 171 glioma samples, were retrieved from TCGA. All the data are available for free online.

### 2.2. ScRNA-Seq Data Analysis

The scRNA-seq data were processed using the Seurat package (v4.3.0) [24]. The quality control criteria are detailed in Section S1.1 in the Supplementary Materials. Quality control included removing cells with a high mitochondrial gene percentage (>20%) or low feature counts. Potential doublets were identified and filtered out using DoubletFinder (v2.0.4). Gene expression was normalized using the ScaleData function. Dimensionality reduction was performed using principal component analysis (PCA), and batch effects were corrected with Harmony. Nonlinear embedding was generated using uniform manifold approximation and projection (UMAP). Cell clusters were annotated by referencing the CellMarker database and validated through automated annotation with SingleR (v2.0.0). Key cell subtypes relevant to glioma were identified based on established marker genes.

### 2.3. ST Data Analysis

The ST data were analyzed in R using the Seurat package (v4.3.0) [25]. The raw unique molecular identifier (UMI) count matrix, along with associated imaging information and spatial coordinates, was loaded and normalized using regularized negative binomial regression (SCTransform). The top 2000 most highly variable genes were selected for PCA, and the first 20 principal components (dims = 1:20) were used for nonlinear dimensionality reduction through UMAP. Clustering was performed using the Louvain algorithm at a resolution of 0.2. Cell-type composition across spatial spots was deconvoluted using the robust cell-type decomposition (RCTD) method, with reference profiles derived from annotated scRNA-seq data and max_cores = 10 for parallel processing [26].

### 2.4. Cell–Cell Communication Analysis

Cell–cell interactions were inferred from the ST data using CellChat (v1.6.1) [27]. Normalized expression matrices and spatially resolved cell subtype annotations were used as input. CellChat identifies significant interactions based on curated ligand–receptor databases and evaluates communication probability via a weighted network model. In this study, standardized single-cell expression data were used as input, in conjunction with the cell subtype information from the ST analysis, to deeply analyze the interactions between cells. Interactions were filtered to retain only those with a *p* < 0.05, minimum interaction strength > 0.05, and at least 10 cells per group (min.cells = 10). The weights and counts of the cell–cell interactions were utilized to assess the strength of these interactions, allowing for the measurement of the activity and potential impact of different cell types in the disease.

### 2.5. Identification of Hub Genes Using Random Survival Forest Analysis

The marker genes of TAFCs (Table S2) were used as candidate genes. The initial filtering criteria were set as logFC > 0.585 and adjusted *p* < 0.05 according to Seurat vignettes and tutorials (https://satijalab.org/seurat/, accessed on 5 June 2025) [28]. A random survival forest (RSF) model was then applied using the randomForestSRC package (v3.5.0) in R to evaluate the prognostic importance of each gene [29]. The analysis was performed with 1000 Monte Carlo iterations. Genes with a relative importance score exceeding 0.4 were selected as definitive hub genes.

### 2.6. Nomogram Model Construction

Nomograms are based on regression analyses [30]. According to the expression of genes and clinical symptoms, line segments with scales are drawn on the same plane according to a certain proportion to express the interaction between variables in the prediction model. By constructing a multi-factor regression model, a score is assigned to each value level of each influencing factor based on its contribution to the outcome variable in the model (the size of the regression coefficient), and then the individual scores are added up to obtain the total score to calculate the predicted value.

### 2.7. Drug Sensitivity Analysis

The chemosensitivity of each tumor sample was forecasted using the R package “oncoPredict”(v1.2) and the Genomics of Drug Sensitivity in Cancer (GDSC) database [31]. Regression analysis was utilized to estimate the IC50 for specific chemotherapeutic agents, and the regression and predictive accuracy were validated using a 10-fold cross-validation on the GDSC training dataset. Throughout this process, the default parameters were used, including the combat method for batch effect correction and the averaging of duplicate gene expression values [32].

### 2.8. Analysis of Relationship Between Immune Cell Infiltration and TAFC Signature Gene Expression

The RNA-seq data from different subgroups of gliomas patients was analyzed using the CIBERSORT algorithm to infer the relative proportions of 22 immune-infiltrating cells and calculate the correlation between gene expression and immune cell content [33].

### 2.9. Regulatory Network Analysis of TAFC Signature Genes

TFs were predicted using the R package “RcisTarget,” (v1.30.0) with computations based on motifs [34]. The normalization enrichment score (NES) for a motif was determined based on the total number of motifs in the database. Additional annotations beyond those annotated by source data were inferred based on motif similarity and gene sequences. The overrepresentation of each motif in a gene set was estimated by calculating the area under the curve (AUC) for each motif–gene set pair, which was derived from the recovery curve of gene set ranking for motifs. The NES for each motif was calculated from the distribution of AUCs across all motifs within the gene set. Key gene-associated miRNAs were obtained from the MiRcode database, and the gene–miRNA network was visualized using Cytoscape software (v3.10.2).

### 2.10. Gene Set Enrichment Analysis

Patients were stratified into high- and low-expression groups based on the expression levels of TAFC signature genes. Gene Set Enrichment Analysis (GSEA) was then applied to analyze the differences in signaling pathways between the groups, using version 7.0 of the MsigDB database as the background gene set [35]. Gene sets with significant enrichment (*p* adj < 0.05) were ranked by consistency score. GSEA is frequently used to explore the biological aspects that are closely related to disease classification. The study was conducted in accordance with the Declaration of Helsinki and approved by the Ethics Committee of the Chinese PLA General Hospital (approval no: 2025-120-S01, date: 12 March 2025).

### 2.11. Gene Set Variability Analysis

Gene Set Variability Analysis (GSVA) is a non-parametric, unsupervised method for assessing the enrichment of transcriptomic gene sets [32]. By scoring gene sets of interest, GSVA translates gene-level changes to pathway-level changes, allowing for the determination of changes in biological functions in samples. In our study, gene sets were downloaded from the Molecular Signatures Database, and GSVA was used to score each set, evaluating potential biological function differences between samples.

### 2.12. Developmental Trajectories of Key Cell Subtypes

Pseudotime analysis was executed using the R package “Monocle3” (v 1.3.1) [36]. Studies at the spatial transcriptome level allow for the characterization of the transcriptional regulation of complex physiological processes and highly heterogeneous cell populations. These studies have led to the discovery of genes that can be used to identify specific cell subtypes, intermediate states of biological processes, and transition states between two different cell fates. In many spatial transcriptome studies, individual cells execute gene expression processes in an asynchronous manner, with each cell reflecting the transcriptional process at a moment in time. Monocle introduces a strategy to sequence individual cells within pseudotime (pseudochronology), taking advantage of the asynchronous processes of individual cells to place them on trajectories corresponding to biological processes such as cell differentiation.

### 2.13. Robustness and Stability Analyses

To ensure the reliability and reproducibility of our findings, we performed robustness checks for critical analytical steps with parameter variations. The detailed methodology and results are provided in Section S1.2 in the Supplementary Materials.

### 2.14. Ethical Approval and Sample Collection

This study was conducted in strict accordance with the Declaration of Helsinki. The study protocol was approved by the Ethics Committee of Air Force Characteristic Medical Center of the Chinese People’s Liberation Army on 12 March 2025 (approval No.: 2025-120-S01). The detailed inclusion criteria for patient enrollment and the specific informed consent process are provided in Section S1.3 in the Supplementary Materials.

### 2.15. HE Staining

Formalin-fixed, paraffin-embedded tissue sections were deparaffinized in xylene and then rehydrated through a graded ethanol series. Nuclei were stained with Harris hematoxylin, followed by differentiation in 1% acid alcohol and bluing in running tap water. Cytoplasmic counterstaining was performed using an eosin Y solution. Finally, the sections were dehydrated through a graded alcohol series, cleared in xylene, and mounted using a resinous medium for microscopic examination.

### 2.16. Immunofluorescence Staining

Paraffin-embedded tissue sections were deparaffinized, rehydrated, and subjected to antigen retrieval in EDTA buffer (pH 9.0) using microwave irradiation. After blocking endogenous peroxidase activity with 3% H_2_O_2_ and non-specific sites with 3% BSA, the sections were incubated overnight at 4 °C with the following primary antibodies: TCF12 (1:200; Proteintech, 14419-1-AP, Wuhan, China), MSTN (1:200; Proteintech, 19142-1-AP, Wuhan, China), and CD163 (1:200; Proteintech, 68218-1-ig, Wuhan, China). After washing, the sections were incubated for 50 min at room temperature with HRP-conjugated secondary antibodies (1:400; SeraCare, 5220-0336 and 5220-0341, MA, USA). Nuclei were counterstained with DAPI (1:4000; Cell Signaling Technology, 4083, MA, USA). Images were acquired using a Zeiss Axio Scope A1 Fluorescence Microscope (Carl Zeiss AG, Oberkochen, Germany) and analyzed with ImageJ software (v1.53).

### 2.17. Statistical Analysis

All statistical analyses were conducted using R language version 4.3.2 (R Foundation for Statistical Computing, Vienna, Austria). Cox regression analysis was used to screen for prognosis-related genes, with the results presented as HRs and 95% CIs. Survival analysis was conducted using the Kaplan–Meier method, and differences between groups were compared using the log-rank test. Comparisons between two groups were performed using the Wilcoxon rank-sum test. All statistical tests were two-sided, and *p* < 0.05 was considered statistically significant.

## 3. Results

### 3.1. Single-Cell and Spatial Transcriptomic Analyses Define Cellular Heterogeneity and Identify TAFCs as a Communication Hub in the Glioma Microenvironment

**Figure 1:**
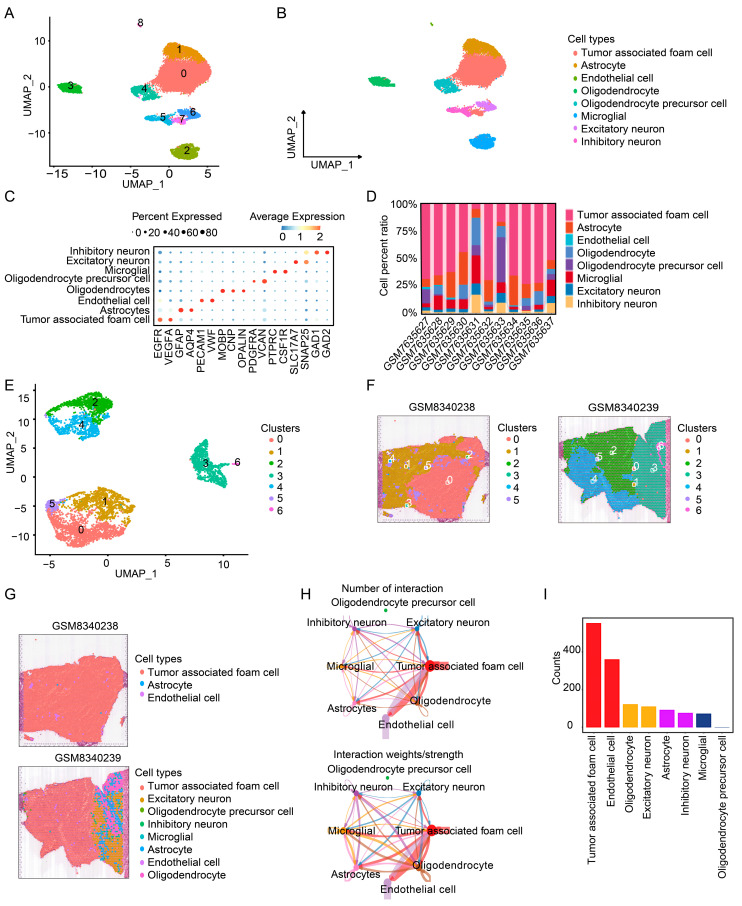
Integrated scRNA-seq and ST analysis reveals cellular heterogeneity and identifies TAFCs as a central interaction hub in glioma. (**A**) UMAP plot depicting the transcriptional landscape of single cells from 11 glioma samples (GSE237673), with distinct colors representing nine different cell clusters, labeled individually as numbers 0 to 8. (**B**) Annotation of eight major cell types on the UMAP plot, including TAFCs (red), astrocytes (brown), endothelial cells (yellow), oligodendrocytes (cyan), oligodendrocyte precursor cells (green), microglia (blue), excitatory neurons (purple), and inhibitory neurons (pink). (**A**) UMAP plot depicting the transcriptional landscape of single cells from 11 glioma samples (GSE237673), with distinct colors representing different cell clusters. (**B**) Annotation of eight major cell types on the UMAP plot, including TAFCs, astrocytes, endothelial cells, oligodendrocytes, oligodendrocyte precursor cells, microglia, excitatory neurons, and inhibitory neurons. (**C**) Heatmap showing the expression levels of canonical marker genes across identified cell types. Color intensity represents average expression levels (red indicates high expression, blue indicates low expression), while dot size indicates the percentage of cells expressing each marker gene within the corresponding cell type. (**D**) Stacked bar plot illustrating the compositional proportions of different cell types across individual samples, demonstrating that TAFCs constitute the most abundant cell population overall. (**E**,**F**) UMAP plots of spatial transcriptomics data from two glioma specimens (GSM8340238 and GSM8340239), with colors indicating seven distinct spatial domains (0–6) identified by the Louvain clustering algorithm. (**G**) Spatial mapping of scRNA-seq-derived cell-type annotations onto tissue sections using the RCTD method, revealing the spatial distribution patterns of different cell populations. (**H**) Heatmap of ligand–receptor interaction strength showing communication weights between different cell types. Redder colors indicate stronger intercellular interactions. (**I**) Network visualization depicting the intercellular communication network between TAFCs and other cell types. Node size represents the number of interactions, while edge thickness indicates communication strength.

A systematic analysis was performed on single-cell transcriptomic data from 11 glioma samples (GSE237673). The initial assessment confirmed significant intercellular transcriptional heterogeneity, providing the basis for subpopulation dissection (Figure S1A). Examination of the UMI count distribution revealed that regions of high transcriptional abundance were primarily associated with epithelial cells (Figure S1B,C). Unsupervised clustering of the scRNA-seq data identified nine transcriptionally distinct cell clusters (Figure 1A). Based on the expression of canonical marker genes, these clusters were annotated as eight major cell types: TAFCs, astrocytes, endothelial cells, oligodendrocytes, oligodendrocyte precursor cells, microglia, excitatory neurons, and inhibitory neurons (Figure 1B). Marker validation confirmed high cell-type specificity; for instance, GFAP expression was predominantly restricted to the astrocytic cluster (Figure 1C). Analysis of cellular composition across integrated samples revealed that TAFCs constituted the most abundant population (Figure 1D). To resolve the spatial architecture, ST data from two glioma sections (GSE270355) were analyzed and clustered into seven spatial domains (Figure 1E,F). RCTD was employed to map the scRNA-seq-derived cell identities onto the ST spots. This spatial deconvolution confirmed the predominant localization of TAFCs within tumor-rich regions (Figure 1G and Figure S1C). The spatial mapping accuracy was further validated by the congruent localization of key cell-type marker genes in the ST data (Figure S1D). Finally, ligand–receptor interaction analysis across the spatial transcriptomics data demonstrated that TAFCs exhibited the highest communication activity. Furthermore, they engaged in the most extensive interaction networks with other cell types in the tumor microenvironment (Figure 1H,I). Collectively, these results delineate the cellular heterogeneity and spatial interaction networks in glioma, establishing TAFCs as a highly connected communication hub within the TME.

### 3.2. Integrated Bioinformatics Analysis Identifies MSTN and TCF12 as Core Prognostic Genes Associated with the Immune Landscape in Glioma

**Figure 2:**
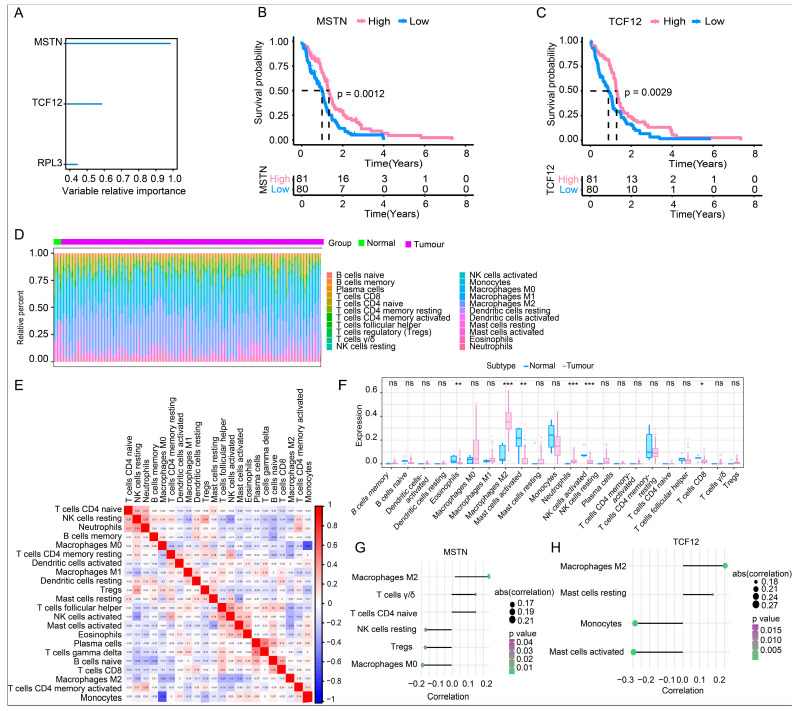
Identification and immune correlates of prognostic genes in TAFCs. (**A**) RSF analysis of TAFC-specific markers identifies three genes (*MSTN*, *TCF12*, and *RPL3*) as top candidates, with values showing variable relative importance > 0.4. (**B**,**C**) Kaplan–Meier survival curves showing that high expression of *MSTN* or *TCF12* is associated with significantly poorer overall survival in glioma patients. The median survival probability differs significantly between the high-expression and low-expression groups. (log-rank test, *MSTN*: *p* = 0.0012; *TCF12*: *p* = 0.0029), whereas RPL3 showed no prognostic relevance. (**D**) Percentage of immune cells in control (green) and glioma (purple) groups. (**E**) Interaction analysis of 22 different immune cells in glioma patients (blue: negative correlation; red: positive correlation). (**F**) Comparisons of immune cells in control (blue) and glioma (purple) groups (*: *p* < 0.05; **: *p* < 0.01; ***: *p* < 0.001; ns: not significant). (**G**) Correlation analysis demonstrates that *MSTN* expression positively correlates with M2 macrophages (correlation > 0.21, *p* < 0.01) and γδ T cells (correlation = 0.17, *p* < 0.05), and negatively correlates with M0 macrophages (correlation = −0.21, *p* < 0.05) and Tregs (correlation = −0.19, *p* < 0.05). (**H**) *TCF12* expression shows a positive correlation with M2 macrophages (correlation = 0.27, *p* < 0.005) and resting mast cells (correlation = 0.18, *p* < 0.05), and a negative correlation with activated mast cells (correlation = −0.24, *p* < 0.005) and monocytes (correlation = −0.27, *p* < 0.005).

To investigate the potential functional role of TAFCs, we analyzed their marker genes. The TCGA glioma cohort was utilized as the study population. Using screening criteria of logFC > 0.585 and adjusted *p* < 0.05, we identified candidate genes and performed RSF analysis to evaluate their prognostic value. Three genes (*MSTN*, *TCF12*, and *RPL3*) showed high relative importance scores > 0.4 (Figure 2A). Survival analysis confirmed that high expression of *MSTN* or *TCF12* was significantly associated with poorer overall survival (OS), whereas RPL3 showed no prognostic relevance (Figure 2B,C). Therefore, *MSTN* and *TCF12* were selected as signature genes for further study. Established clinical prognostic variables (gender and age) together with *MSTN* and *TCF12* expression levels (high- and low-expression groups) were analyzed using univariate and multivariate Cox regression models. Univariate analysis revealed that high *MSTN* expression was associated with poor prognosis (HR = 1.251, 95% CI: 1.094–1.432, *p* = 0.001), and high *TCF12* expression also conferred increased risk (HR = 1.530, 95% CI: 1.196–1.957, *p* < 0.001) (Figure S2A,C). Multivariate analysis further confirmed that, after adjusting for gender and age, *MSTN* (HR = 1.245, 95% CI: 1.085–1.429, *p* = 0.002) and *TCF12* (HR = 1.603, 95% CI: 1.205–2.012, *p* < 0.001) both remained independent adverse prognostic factors for glioma, with *TCF12* demonstrating greater prognostic value (Figure S2B,D). These results were consistent with the Kaplan–Meier survival analysis.

Next, we analyzed their association with the immune microenvironment. Analysis of 22 immune cell subsets revealed distinct infiltration patterns in tumor samples, including increased M2 macrophages and neutrophils, and decreased eosinophils, activated mast cells, activated NK cells, and follicular helper T cells (Figure 2D–F). Correlation analysis indicated that both MSTN and *TCF12* expression positively correlated with M2 macrophage infiltration and showed distinct associations with other immune subsets. *MSTN* was positively linked to γδ T cells and negatively correlated with M0 macrophages and Tregs (Figure 2G). *TCF12* correlated positively with resting mast cells and negatively correlated with activated mast cells and monocytes (Figure 2H). These correlative findings suggest a potential association between *MSTN* and *TCF12* and an immunosuppressive tumor microenvironment that may involve M2 macrophage recruitment or polarization. Furthermore, the expression of both genes showed negative correlations with multiple chemokines (e.g., CCL2 and CCR7), MHC-related molecules (e.g., TAPBP), and immunomodulators (e.g., TGFB1 and CD70), but positive correlations with CCL25, TAP1, VTCN1, and TNFRSF13C (Figure S3A–E). Collectively, these results indicate that *MSTN* and *TCF12* may be closely associated with immune cell recruitment and functional polarization, likely contributing to immunosuppression in glioma through coordinated regulation of specific immune factors.

### 3.3. Co-Expression Network of Key Genes and Disease-Associated Genes in Single Cells and Functional Differences in Metabolic Pathways

**Figure 3:**
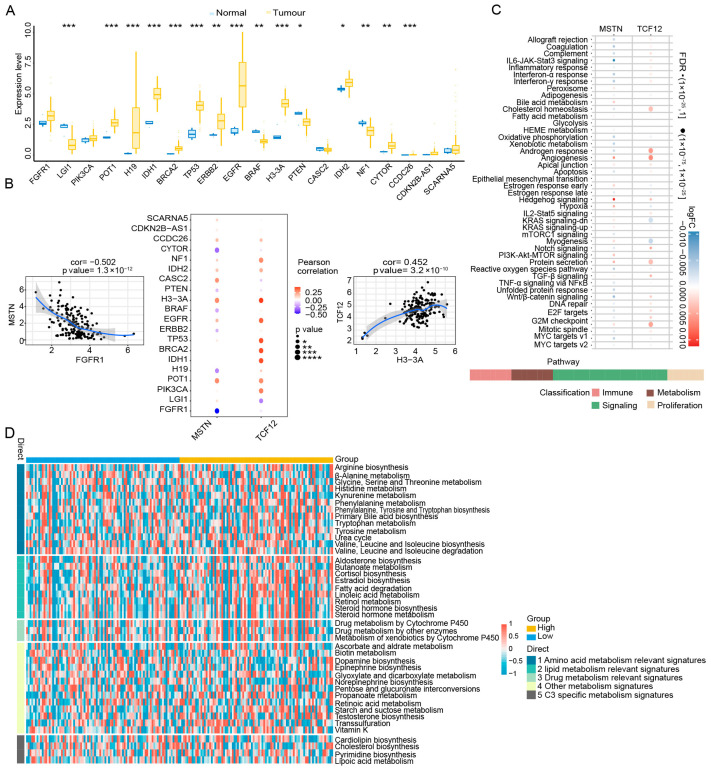
Associations of *MSTN* and *TCF12* with disease-related genes and immune–metabolic pathway activities. (**A**) Expression profiles of the top 20 glioma-related genes (ranked by transcription levels from GeneCards), highlighting differentially expressed genes such as LGI1, POT1, and H19 (blue: control group; yellow: tumor group). (**B**) Bubble map for the Pearson correlations between *MSTN* and *TCF12* and disease-related genes. Larger circles indicate that the *p*-value is closer to zero; a redder color indicates a stronger positive correlation and a deeper purple indicates a stronger negative correlation. (**C**) Single-cell pathway activity scores indicating elevated activity of *MSTN* and *TCF12* in immune–metabolic pathways, including cholesterol homeostasis, androgen response, and angiogenesis. A larger dot size indicates a higher enrichment significance (FDR closer to 0); logFC represents fold change; red indicates a positive correlation, and blue indicates a negative correlation. (**D**) Single-cell metabolic pathway activity associated with *MSTN* and *TCF12* expression. *Y*-axis: 5 major metabolic signatures including amino acid, lipid, drug, other metabolism, and C3-specific pathways. Red indicates high pathway activity; blue indicates low pathway activity. *X*-axis: yellow = high gene expression group; cyan = low gene expression group. *: *p* < 0.05; **: *p* < 0.01; ***: *p* < 0.001; ****: *p* < 0.0001.

**Figure 4:**
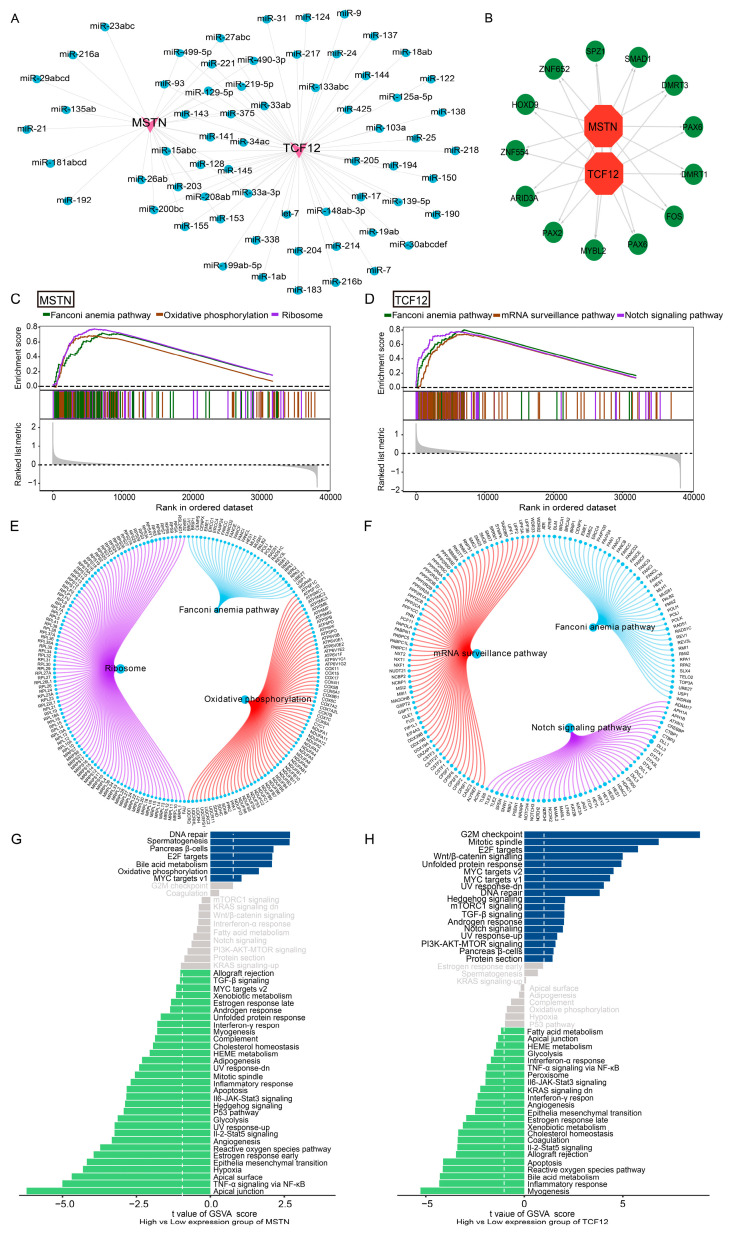
Transcriptional and post-transcriptional regulatory networks of *MSTN* and *TCF12*, and their association with functional pathways. (**A**) miRNA–mRNA interaction network predicted by the MiRcode database showing 63 miRNAs that could potentially regulate *MSTN* and *TCF12*. Key miRNAs such as miR-129-5p, miR-219-5p, and miR-221 are highlighted. (**B**) Transcriptional regulatory network illustrating TFs enriched in the promoters of *MSTN* and *TCF12*. The most significantly enriched motif is cisbp_M1404 (NES = 5.82). (**C**–**F**) GSEA reveals pathways significantly associated with *MSTN* (ribosome, oxidative phosphorylation, Fanconi anemia) and *TCF12* (mRNA surveillance, Notch signaling, Fanconi anemia). (**G**,**H**) GSVA highlights distinct functional profiles: *MSTN* correlates with DNA repair and spermatogenesis pathways, while *TCF12* is associated with G2/M checkpoint and E2F target pathways.

To explore the functional context of MSTN and *TCF12*, we analyzed their association with glioma-related genes from the GeneCards database. The expression of the top 20 disease-related genes based on transcription levels, including LGI1, POT1, and H19, differed significantly between healthy and tumor tissues (Figure 3A). Correlation analysis further revealed that *TCF12* expression positively correlated with H3-3A (r = 0.452) and *MSTN* expression negatively correlated with FGFR1 (r = −0.502) (Figure 4B). Single-cell co-expression networks constructed between *MSTN* or *TCF12* and the top glioma-associated genes indicated potential regulatory interactions (Figure S4A–F). Pathway activity analysis using AUCell demonstrated that cells with high expression of *MSTN* or *TCF12* exhibited elevated scores in immune–metabolic pathways, notably cholesterol homeostasis, androgen response, and angiogenesis (Figure 3C). When cells were stratified based on immune-infiltration scores, the high-expression group showed reduced activity of lipid–metabolism signatures (Figure 3D), suggesting that lipid metabolic remodeling is linked to the immunomodulatory function of these genes in the glioma microenvironment.

### 3.4. Predicted Regulatory Networks and Pathways of Two Signature Genes

To further explore the potential regulatory landscape of *MSTN* and *TCF12*, we performed bioinformatic analyses to characterize their predicted associations with non-coding RNAs and transcription factors (TFs). Reverse prediction using the MiRcode database identified 63 potential miRNAs that interact with *MSTN* or *TCF12*, yielding 82 predicted mRNA–miRNA pairs (Figure 4A), including miR-129-5p, miR-219-5p, and miR-221, suggesting possible post-transcriptional regulation within the TME. Concurrently, TF motif enrichment analysis was conducted on the promoter regions of gene sets associated with *MSTN* and *TCF12* using RcisTarget (v1.30.0). Multiple TF motifs exhibited significant enrichment, with the highest enrichment (NES = 5.82) observed for the cisbp_M1404 motif (Figure 4B; Table S3). These results indicate that *MSTN* and *TCF12* are embedded within transcriptional programs characterized by specific TF motif enrichment patterns.

Functional pathway enrichment was assessed using GSEA and GSVA. GSEA showed that *MSTN* was notably enriched in ribosome, oxidative phosphorylation, and Fanconi anemia pathways (Figure 4C–E), whereas *TCF12* was enriched in mRNA surveillance, Notch signaling, and Fanconi anemia pathways (Figure 4D–F). GSVA further highlighted distinct functional profiles. *MSTN* correlated with DNA repair and spermatogenesis pathways (Figure 4G), while *TCF12* associated strongly with G2/M checkpoint and E2F target pathways (Figure 4H). These results imply that *MSTN* and *TCF12* contribute to glioma progression by modulating fundamental processes such as cell-cycle progression, DNA repair, and metabolic pathways, thereby shaping the immune-functional landscape of the TME.

### 3.5. Expression Profiles and Developmental Trajectories of Key Genes in ST Data

**Figure 5:**
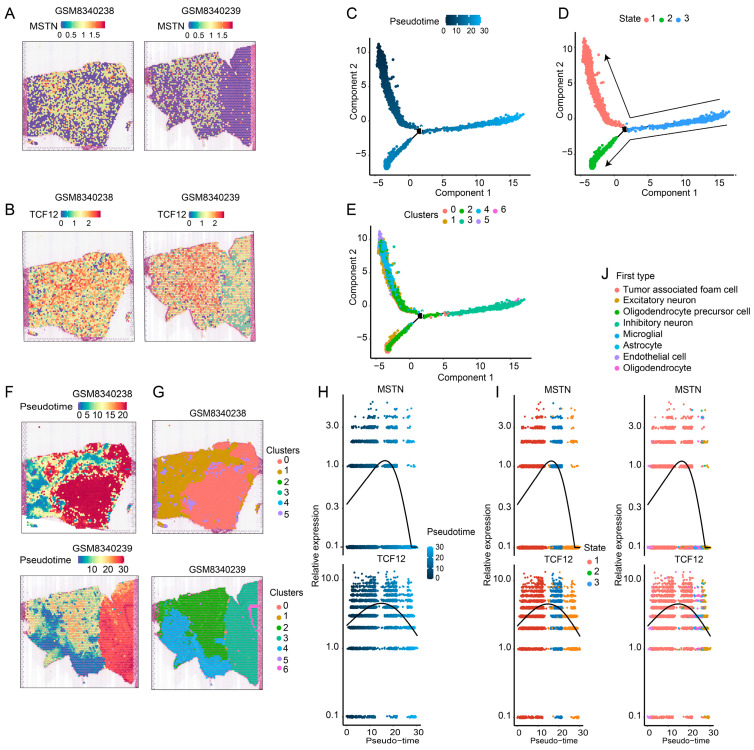
Spatial heterogeneity and differentiation-associated expression dynamics of *MSTN* and *TCF12*. (**A**,**B**) Spatial distribution of *MSTN* and *TCF12* expression in two independent glioma samples (GSM8340238 and GSM8340239) from the GEO database. Color scale represents normalized *MSTN* and *TCF12* expression levels, with blue indicating low expression and red indicating high expression. (**C**) Pseudotime trajectories displayed in component space. The color gradient from dark blue to light yellow represents the pseudotime value, with dark blue indicating the start of the trajectory (early differentiation) and light yellow indicating the end (late differentiation). (**D**) Distribution of cell states (State 1, 2, 3) in component space, with distinct colors representing different cell states. (**E**) Distribution of cell clusters (Clusters 0–6) in component space, with distinct colors representing seven different cell clusters. (**F**,**G**) Projection of pseudotime trajectories onto corresponding HE-stained sections enables dual-dimensional “pseudotime-spatial” visualization, with different colors representing the clustered cell clusters. (**H**) Expression dynamics of MSTN and TCF12 along the pseudotime axis. Each colored line represents the relative expression level of MSTN (left) and TCF12 (right) for a specific cell type, with colors corresponding to the cell types defined in (**J**). (**I**) Expression dynamics of MSTN and TCF12 along the pseudotime axis across three cell states (State 1, 2, 3). The color gradient from purple to yellow represents the pseudotime value, with purple indicating early pseudotime and yellow indicating late pseudotime. Each point represents a cell, colored by its state: State 1 (red), State 2 (blue), and State 3 (green). (**J**) Cell type annotation legend showing the eight major cell types identified, with distinct colors corresponding to each cell type: Tumor associated foam cell (red), Excitatory neuron (blue), Oligodendrocyte precursor cell (green), Inhibitory neuron (purple), Microglial (orange), Astrocyte (yellow), Endothelial cell (cyan), and Oligodendrocyte (pink).

ST analysis revealed distinct expression patterns of *MSTN* and *TCF12* across glioma tissues. Integration of Visium maps from two independent samples demonstrated significant spatial heterogeneity, with both genes showing region-specific expression gradients between the tumor core, invasive margin, and adjacent non-tumor regions (Figure 5A,B), indicating microenvironment-driven transcriptional regulation of these genes. To resolve their dynamic expression along cellular differentiation trajectories, we performed pseudotime analysis by integrating the scRNA-seq and ST data. Pseudotime trajectories were reconstructed based on transcriptomic similarity (Figure 5C–E) and spatially mapped onto corresponding HE-stained sections, enabling dual-dimensional pseudotime–spatial visualization (Figure 5F,G). Expression dynamics of *MSTN* and *TCF12* were then quantified along the pseudotime axis across nine annotated cell types. The results demonstrated clear, cell-type-dependent expression patterns for both genes (Figure 5H,I), suggesting that they participate in the regulation of cell-state transitions and tissue organization during glioma progression.

### 3.6. Prediction of Drug Sensitivity and Prognosis Based on Expression of Key Genes

**Figure 6:**
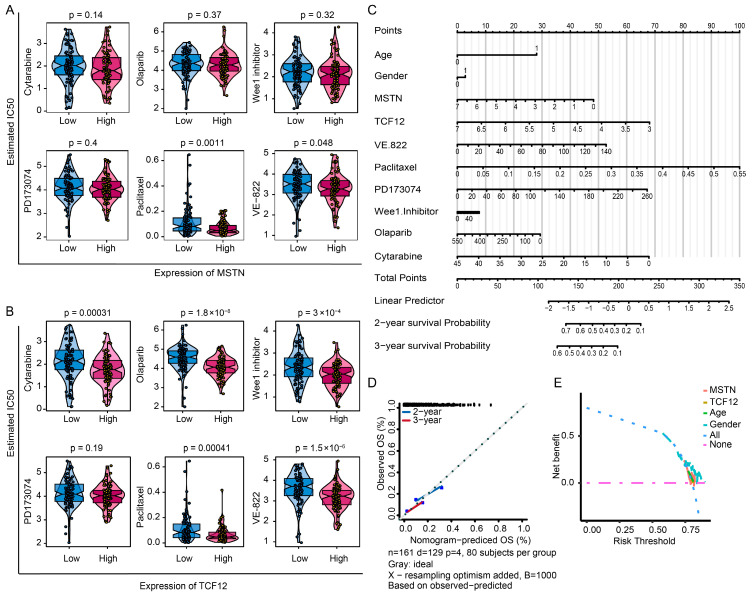
Association of *MSTN* and *TCF12* with chemotherapy sensitivity and a clinical prognostic nomogram. (**A**,**B**) Correlation analysis between expression of *MSTN* or *TCF12* and predicted sensitivity to common chemotherapeutic agents from the GDSC database. *MSTN* expression correlates with sensitivity to paclitaxel (*p* = 0.0011) and VE-822 (*p* = 0.048), while *TCF12* expression is associated with sensitivity to cytarabine (*p* = 0.00031), olaparib (*p* = 1.8 × 10^−8^), a WEE1 inhibitor (*p* = 3 × 10^−4^), paclitaxel (*p* = 0.00041), and VE-822 (*p* = 1.5 × 10^−6^). (**C**) Prognostic nomogram integrating *MSTN* and *TCF12* expression with clinical variables (age, gender, chemotherapy regimen). (**D**) Nomogram calibrated for 2- and 3-year overall survival prediction for TCGA glioma patients. (**E**) Decision curve analysis of prediction models (net benefit across risk thresholds (0–1)). Blue: combined model; red: *MSTN*; yellow: *TCF12*; green: age; cyan: gender; gray: all. The combined model demonstrates the greatest clinical utility in the 0.3–0.7 threshold range.

To assess the potential translational relevance of *MSTN* and *TCF12*, we analyzed their association with chemotherapeutic responses using drug sensitivity data from the GDSC database and the R package “oncoPredict.” The agents evaluated, including cytarabine, olaparib, a Wee1 inhibitor, PD173074, paclitaxel, and VE-822, were selected based on their established or investigational relevance to glioma therapy [37,38,39,40,41,42,43]. *MSTN* expression was predicted to be significantly correlated with sensitivity to paclitaxel and VE-822. *TCF12* expression was potentially associated with sensitivity to cytarabine, olaparib, a Wee1 inhibitor, paclitaxel, and VE-822 (Figure 6A,B).

Logistic regression analysis revealed that clinical parameters and the two significant genes (*MSTN* and *TCF12*) contributed to glioma risk stratification. Higher composite scores were associated with poorer 2- and 3-year survival outcomes (Figure 6C). At the same time, the calibration curves further demonstrated excellent agreement between nomogram-predicted and observed overall survival probabilities (Figure 6D,E).

### 3.7. Upregulation and Macrophage Co-Localization of Two Signature Genes in Glioma Tissues Suggests a Regulatory Role in the TME

**Figure 7:**
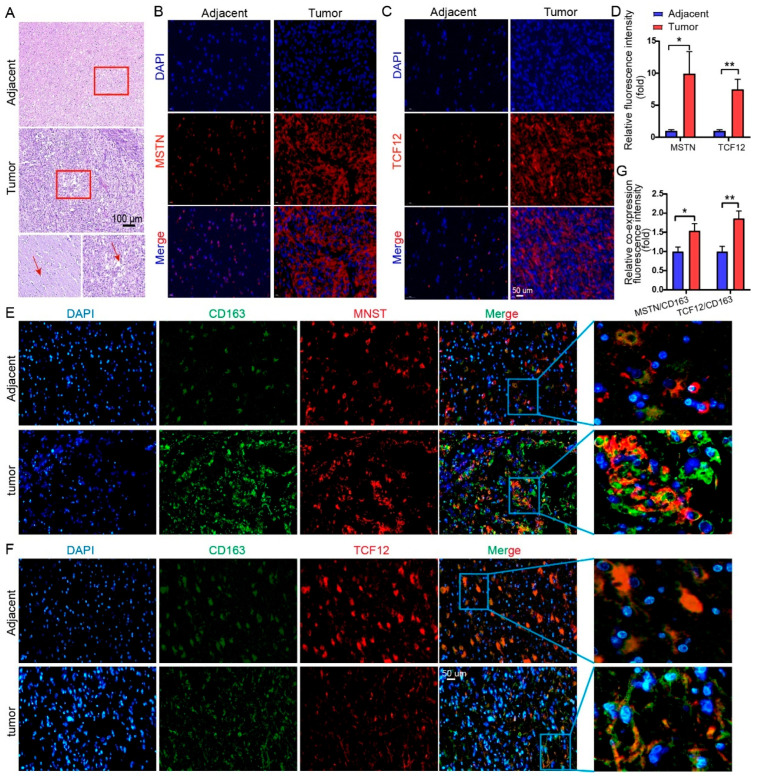
Histological and immunofluorescence validation of MSTN and TCF12 expression in TAFC-enriched glioma tissues. (**A**) Representative HE staining of paired peritumoral and glioma tissues. Peritumoral regions show an abundance of microglia, while tumor areas are enriched with foam-like cells The two smaller panels below are magnified views of the areas outlined by the red boxes. Red arrows indicate macrophages.. (**B**,**C**) Immunofluorescence staining confirms significant upregulation of MSTN (**B**) and TCF12 (**C**) in glioma compared to adjacent tissues. (**D**) Quantitative analysis of MSTN and TCF12 fluorescence intensity. (**E**,**F**) Dual-color immunofluorescence co-staining of macrophage marker CD163 and MSTN (**E**) and TCF12 (**F**). Yellow signal indicates co-localization, which is markedly increased in tumor tissues. The right panel shows a magnified view of the blue box. (**G**) Quantification of the percentage of CD163^+^ macrophages co-positive for MSTN or TCF12. Nuclei are counterstained with DAPI. Green: CD163; red: MSTN or TCF12; blue: DAPI. Data is presented as mean ± SEM (*n* = 5). Scale bars = 50 μm. *: *p* < 0.05; **: *p* < 0.01.

Previous reports suggest TAFCs may arise from lipid-laden tumor-associated macrophages [25,30]. HE staining revealed adjacent non-tumor tissue containing an abundance of microglia, while tumor regions were enriched with foam-like cells (Figure 7A). Next, we performed immunofluorescence analysis on five paired glioma and adjacent non-tumor tissue samples. Immunofluorescence confirmed that both MSTN and TCF12 were significantly upregulated in tumor tissues compared to adjacent regions (Figure 7B–D). Dual-color co-staining with the macrophage marker CD163 and MSTN or TCF12 demonstrated a markedly increased proportion of CD163^+^ macrophages co-expressing either gene within tumor areas (Figure 7E–G). These results provide histological and protein-level validation that MSTN and TCF12 are elevated in TAFC-enriched regions and are co-localized with tumor-associated macrophages, supporting their potential role in mediating macrophage-dependent immune remodeling in the glioma microenvironment.

## 4. Discussion

In glioma, identifying TME cell subsets associated with clinical prognosis is crucial for developing novel biomarkers and therapeutic strategies [19,44,45]. In this study, by integrating single-cell and spatial transcriptomic technologies, we systematically delineated the cellular composition and spatial interaction network of the glioma TME, and identified TAFCs as a prominent, spatially distinct, and highly connected active core within the TME. Further investigation revealed that the expression levels of TAFC signature genes were significantly associated with poor prognosis and chemotherapy resistance in glioma patients, underscoring their clinical relevance. These signature genes may be associated with the formation of an immunosuppressive TME and malignant progression of glioma through processes involving immune cell infiltration, particularly macrophage polarization toward an M2 phenotype, remodeling of lipid metabolism-related pathways, and changes to multi-layer transcriptional and post-transcriptional networks.

Macrophages within the TME, as highly plastic innate immune cells, can differentiate into functionally diverse phenotypes in response to local signals, playing an important role in tumor progression [46,47,48]. Among them, TAMs, as a major component of the TME, have been widely demonstrated to promote tumor cell proliferation, angiogenesis, lymphangiogenesis, invasion, and metastasis, and mediate therapeutic resistance, making them a highly promising therapeutic target [45,49,50,51,52]. In recent years, a special subset of TAMs, TAFCs, has attracted attention due to their unique lipid metabolic phenotype. Foam cells have been identified in multiple types of cancer tissues, including glioma, prostate cancer, colorectal cancer, papillary renal cell carcinoma, and other solid tumors [53,54,55,56]. The concept of foam cells originate from atherosclerosis, and refers to the foam-like cells that macrophages transform into after engulfing large amounts of lipids [57,58,59]. These cells are not only involved in lipid metabolism and inflammatory responses, but also play important roles in immune regulation, tissue repair, and tumor progression. In glioblastoma, recent evidence indicates that tumor cells can drive the transformation of TAMs into TAFCs by releasing extracellular vesicles that transfer lipids [60,61,62]. Furthermore, one study found that lipid droplet-laden TAMs in glioma can undergo diacylglycerol O-acyltransferase 1 (DGAT1)-dependent lipid metabolic reprogramming, secreting lipid mediators such as oxidized phospholipids, which directly stimulate the self-renewal, proliferation, and invasive capacity of glioma stem cells [22]. This finding directly links the metabolic properties of TAFCs to their tumor-promoting functions, suggesting that they have an important role in maintaining the stemness of tumor cells. Beyond glioma, TAFCs also exhibit clear tumor-promoting functions in other solid tumors. For example, TAMs in prostate cancer can undergo MAFB/TREM2-dependent lipid metabolic reprogramming, taking up and processing lipids and subsequently transferring oxidized lipids to tumor cells, thereby activating the PI3K-AKT-mTORC1 signaling axis and driving tumor progression [63]. In papillary renal cell carcinoma, tumor cells secrete Chemerin, IL-8, and CXCL16 to cooperatively recruit monocytes and induce their differentiation into lipid-accumulating foam-like macrophages within the tumor. This macrophage subset is associated with high-grade tumors [53]. These studies collectively indicate that TAFCs may reshape the immunosuppressive microenvironment through conserved or tissue-specific metabolic–immunoregulatory mechanisms, thereby promoting tumor progression in different cancer types.

This study suggests that *TCF12* and *MSTN* could serve as a TAFC-specific prognostic signature in glioma associated with lipid metabolic reprogramming with immunosuppressive macrophage polarization. Unlike conventional TAM markers that broadly associate with M2 status, high *MSTN*/*TCF12* expression specifically marks the TAFC subset and is significantly associated with poor prognosis and chemotherapy resistance, offering a refined tool for patient stratification with important clinical implications. The multi-omics analyses suggest an association of these genes with a “metabolic–immune” tumor-promoting phenotype in glioma. High *MSTN*/*TCF12* expression correlated with M2 macrophage infiltration, neutrophil recruitment, and elevated immune checkpoint molecules (IL-10, CD274, CD244, VTCN1, BTLA, KDR), which are all associated with T-cell activity and promote immune evasion. This association of intrinsic lipid metabolic dysregulation with local immunosuppression may reflect a process functionally distinct from broad M2 polarization. Our findings exhibit both convergence with and divergence from the established roles of these genes in other malignancies. For instance, *MSTN* is characterized as a systemic cachexia mediator in gastric, lung, and leukemic cancers, where its upregulation correlates with muscle wasting and subsequent poor prognosis [64]. However, a multi-omics analysis suggested that it is involved in local TME processes in glioma through macrophage-mediated immunosuppression independent of systemic muscle atrophy. Similarly, *TCF12* has been reported to promote hepatocellular carcinoma progression through CXCR4-mediated MAPK/ERK and PI3K/AKT signaling [65]; however, the analysis suggests that *TCF12* may also be implicated in glioma progression through CXCR4 or other immune regulatory factor-mediated lipid metabolic–immune crosstalk pathways. This context-dependent functional profile suggests that canonical cancer genes within specialized TAM subpopulations should be studied rather than in bulk tumor analyses. Collectively, our findings suggest a different role for *MSTN*/*TCF12* in local immunometabolic microenvironments in glioma compared to conventional oncogenic contexts. This distinction carries immediate therapeutic implications: while anti-*MSTN* therapies in other cancers target muscle preservation, glioma-specific interventions could focus on disrupting TAFC-mediated immunosuppressive TMEs. The cooperative expression of *MSTN* and *TCF12* in our study indicates that they are a potential therapeutic target, offering superior specificity over broad TAM depletion strategies. Future studies could further validate the potential synergy with immune checkpoint inhibitors or temozolomide-based chemotherapy.

We acknowledge several limitations of this study. First, the sample size (eleven samples for scRNA-seq and two samples for ST) may be insufficient to capture the full spectrum of biological heterogeneity within the glioma patient population, and certain rare cell subtypes with significant biological or clinical relevance may not have been adequately characterized. Second, the limited sample size precluded robust stratified analyses across specific molecular subtypes (e.g., IDH-mutant versus IDH-wildtype) or tumor grades, preventing us from addressing whether cellular states correlate with distinct molecular drivers or whether spatial organization varies by tumor grade. Third, our findings should be interpreted as preliminary and hypothesis-generating rather than conclusive, and their generalizability to the broader glioma population is limited. Validation in larger, multi-center independent cohorts is therefore imperative. Future studies should incorporate substantially larger sample sizes with balanced distributions of key clinicopathological characteristics, including molecular subtype, tumor grade, patient age, and treatment history, to enable more comprehensive and statistically powered analyses. To build upon the current findings, subsequent research will focus on elucidating the functional roles of *MSTN* and *TCF12* within the glioma microenvironment.

## 5. Conclusions

This integrated multi-omics study identified TAFC as the most abundant and centrally connected cellular subset in the glioma tumor microenvironment, with *MSTN* and *TCF12* identified as the key core TAFC genes that drive the immunosuppressive microenvironment and metabolic reprogramming, thereby promoting glioma progression and poor prognoses. The relationships between these signature genes and M2 macrophage polarization, neutrophil recruitment, immune checkpoint molecules, chemotherapy sensitivity, and multi-layered regulatory networks were systematically elucidated through single-cell deconvolution, spatial transcriptomics, and bioinformatic analysis of bulk RNA-seq data. Furthermore, a prognostic nomogram was constructed by integrating clinical information and TAFC signature genes, which demonstrated accurate prediction of patient survival and therapeutic responses. Hence, this preliminary yet relatively comprehensive study provides a novel perspective for understanding the spatial heterogeneity of TAMs in the glioma microenvironment, and *MSTN* and *TCF12* may serve as promising biomarkers with potentially actionable molecular mechanisms.

## Data Availability

The data supporting the findings of this study can be accessed through the public databases GEO (https://www.ncbi.nlm.nih.gov/geo/, accessed on 20 June 2025) and TCGA (https://portal.gdc.cancer.gov/, accessed on 23 June 2025). The use of data from public databases was performed in compliance with the terms of use of those databases. The raw data supporting the conclusions of this article will be made available by the authors, without undue reservation.

## Supplementary Materials

The following supporting information can be downloaded at: https://www.mdpi.com/article/10.3390/cimb48030289/s1.

## Abbreviations

The following abbreviations are used in this manuscript:

TAFC	tumor-associated foam cells
TME	tumor microenvironment
*MSTN*	myostatin
*TCF12*	transcription factor 12
RPL3	ribosomal Protein L3
RSF	random survival forest
RCTD	robust cell-type decomposition
GSEA	gene set enrichment analysis
GSVA	gene set variability analysis
TFs	transcriptional factors
HR	hazard ratio
CI	confidence interval
