# Supplementary material for: MSTN and TCF12 as Candidate Immunometabolic Signatures in Glioma-Associated Foam Cells: Insights from Integrated Multi-Omics Analysis
Source: Curr Issues Mol Biol. 2026 Mar 9;48(3):289. doi: 10.3390/cimb48030289 (PMC13025612; doi:10.3390/cimb48030289)
Supplement: Supplementary file 1 [file cimb-48-00289-s001.zip › Supplementary Figures S1-S4.pdf]

Supplementary Materials

MSTN and TCF12 Emerge as Master Immunometabolic Regulators in Glioma-Associated Foam Cells from Integrated Multi-Omics Analysis

Xu Liu [1], Zhuo Song [1], Zhijia Sun [1], Chen Liu [1], Xiaoli Kang [1], Huilian Qiao [2], Xinzhuo Tu [2], Teng Li [2], Zhiguang Fu [1,*] and Yingjie Wang [1,*]

[1] Department of Radiotherapy Oncology, Air Force Medical Center, PLA, Air Force Medical University, Beijing 100142, China; xuxu_lh@163.com (X.L.); drzhuosong2013@126.com (Z.S.); sunzhijia301@163.com (Z.S.); liuchen_af@fmmu.edu.cn (C.L.); kangxioali-001@163.com (X.K.)

[2] Department of Pathology, Air Force Medical Center, PLA, Air Force Medical University, Beijing 100142, China; qiaohi330@126.com (H.Q.); pathologytu@163.com (X.T.); jacksonlt@163.com (T.L.)

* Correspondence: fuzhiguang@fmmu.edu.cn (Z.F.); wangyingjie666@fmmu.edu.cn (Y.W.)

Materials and Methods ~~and Materials~~

1.1. ScRNA-seq Data Analysis

Quality control was performed to exclude low-quality cells and potential doublets based on the following criteria. Mitochondrial gene percentage (percent.mt) ≤ 20.16244%, ribosomal gene percentage (percent.ribo) ≤ 0.737722%, number of detected genes (nFeature_RNA) between 200 and 5,268.405, and total UMI counts (nCount_RNA) ≤ 10,177.51. Potential doublets were identified and filtered out using DoubletFinder (v2.0.4) with pK parameter optimization via the bcmvn method and an expected doublet formation rate of 7.5%.

1.2. Robustness and Stability Analyses

Clustering resolution optimization: To determine the optimal clustering resolution, we systematically tested values ranging from 0.1 to 0.8 using the Seurat FindClusters function with default parameters. For each resolution, we assessed: (i) the number of clusters obtained, (ii) silhouette scores for cluster cohesion and separation, and (iii) biological interpretability of marker genes. Resolutions 0.4–0.6 yielded consistent identification of nine major cell populations, including the TAFC cluster characterized by APOE and TREM2 expression. Resolution 0.2 was ultimately selected to achieve finer granularity for subpopulation identification while maintaining cluster stability.

RSF hyperparameter sensitivity analysis: We performed a grid search of RSF hyperparameters to evaluate the stability of variable importance rankings. The parameter space included mtry ∈ {2, 3, 4, 5} and nodesize ∈ {5, 10, 15, 20}, resulting in 16 unique configurations. For each configuration, we computed permutation-based variable importance scores and ranked genes by relative importance. MSTN and TCF12 demonstrated remarkable stability, ranking within the top 3 variables in 15/16 and 16/16 configurations, respectively. This consistency confirms their robust prognostic significance independent of specific hyperparameter choices.

Batch correction validation: Pre-integration UMAP visualization revealed clear sample-specific clustering, indicating substantial batch effects. Post-Harmony integration, cells clustered by cell type rather than sample origin, with batch mixing scores improving from 0.42 to 0.89. Cell type-specific marker expression patterns were preserved following correction, confirming that biological variation was maintained while technical variation was removed.

Differential expression robustness: We tested three alternative logFC thresholds (0.25, 0.5, 1.0) for identifying TAFC marker genes. At all thresholds, MSTN and TCF12 exhibited significant upregulation in TAFCs compared to other myeloid cells (adjusted $p < 0.05$, Wilcoxon rank-sum test). Effect sizes (Cohen's d) were 1.45, 1.38, and 1.22 for MSTN, and

1.38, 1.31, and 1.15 for TCF12 at thresholds 0.25, 0.5, and 1.0, respectively, indicating consistent differential expression across stringency levels.

1.3. Sample Collection and Informed Consent

Inclusion criteria were: (1) pathologically confirmed glioma; (2) age ≥18 years; (3) full capacity for civil conduct. Exclusion criteria were: (1) previous radiotherapy or chemotherapy; (2) concomitant other malignancies. A total of 5 paraffin-embedded tissue samples (tumor and paired adjacent non-tumor tissues) were collected from March 2022 to March 2025. All samples were obtained from patients who underwent surgical resection at our hospital (Air Force Characteristic Medical Center, Chinese People's Liberation Army). Researchers explained the study purpose, sample usage, potential risks, and privacy protection measures to all patients before sample collection. Written informed consent was obtained from all participants. Patients were informed of their right to withdraw consent at any time without affecting their medical treatment. All samples were de-identified using a coding system. Direct personal identifiers (name, medical record number, etc.) were removed, and only essential clinical-pathological information (age, gender, pathological grade) was retained. Paraffin blocks were stored at room temperature in a secured facility with restricted access limited to authorized researchers.

Supplementary Figures and Legends

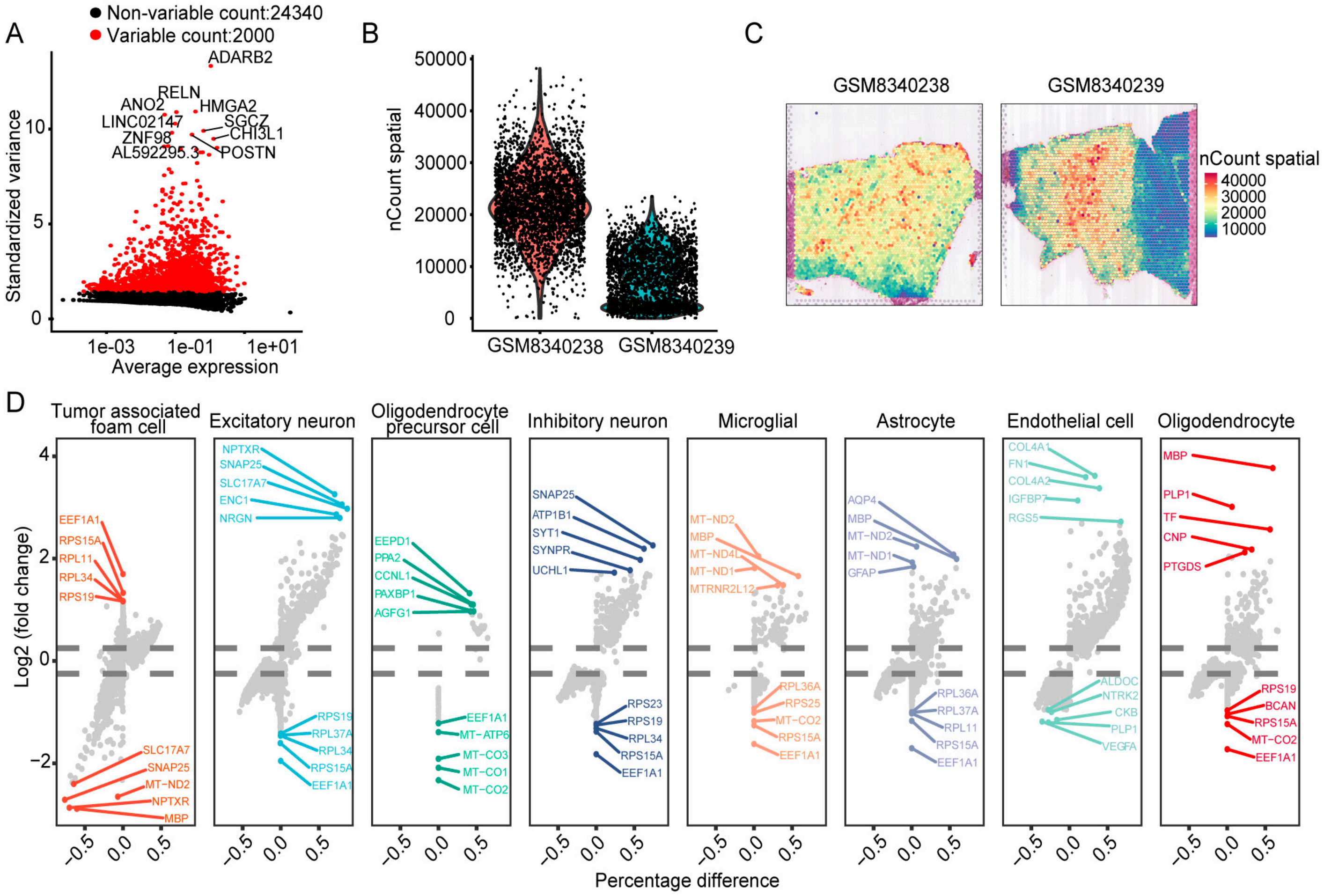

Figure S1. Genes with intercellular differences. (**A**) Single-cell transcriptomic data reveals significantly variable genes among cells. (**B**,**C**) Through the distribution of UMI counts, we observed that regions with high UMI counts are typically associated with epithelial cells. (**D**) Representative marker genes for each subpopulation.

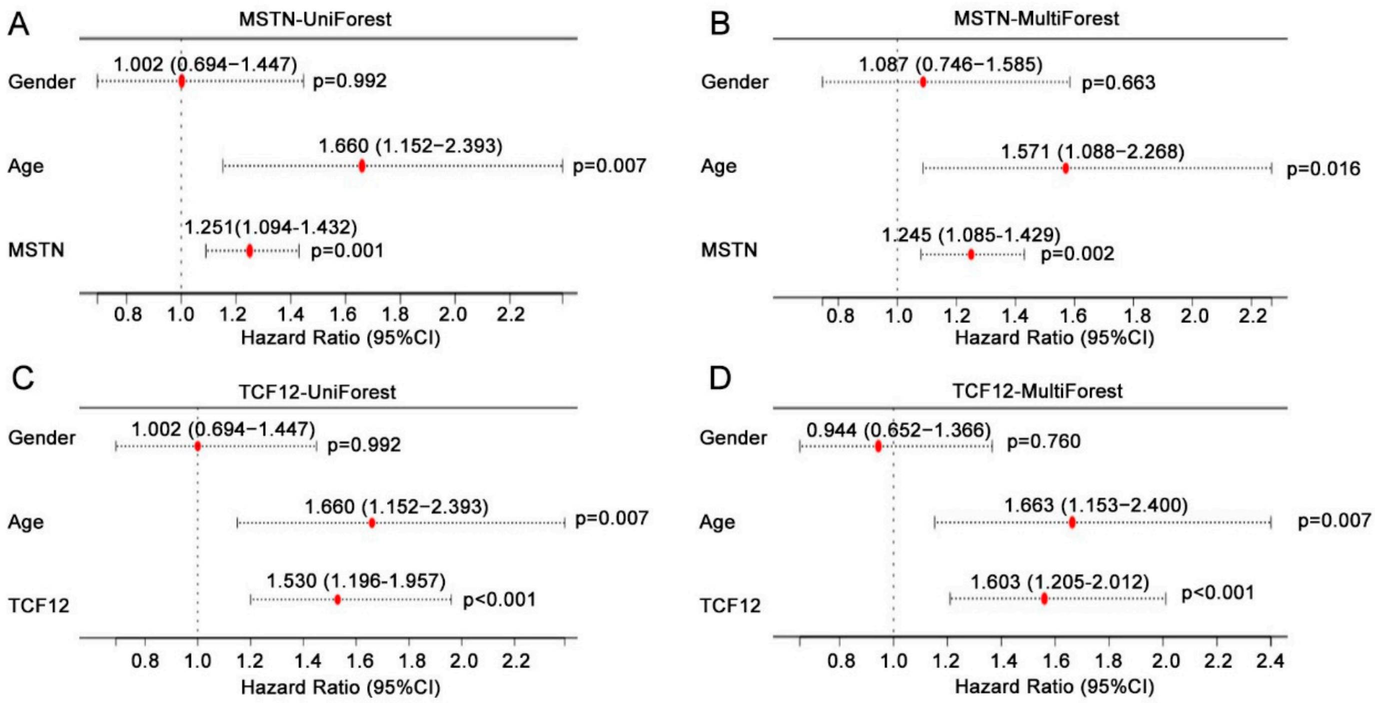

Figure S2. MSTN and TCF12 are independent predictive prognostic factors in TCGA glioma cohort. (**A**) Univariate Cox regression analysis of MSTN expression and clinical variables. High MSTN expression is significantly associated with poor prognosis (HR = 1.251, 95% CI: 1.094-1.432, p = 0.001). (**B**) Multivariate Cox regression analysis of MSTN. After adjusting for gender and age, high MSTN expression remains an independent adverse prognostic factor (HR = 1.245, 95% CI: 1.085-1.429, p = 0.002). (**C**) Univariate Cox regression analysis of TCF12 expression and clinical variables. High TCF12 expression confers increased risk (HR = 1.530, 95% CI: 1.196-1.957, p < 0.001). (**D**) Multivariate Cox regression analysis of TCF12. TCF12 remains an independent adverse prognostic factor after adjustment (HR = 1.603, 95% CI: 1.205-2.012, p < 0.001), demonstrating stronger prognostic value than MSTN.

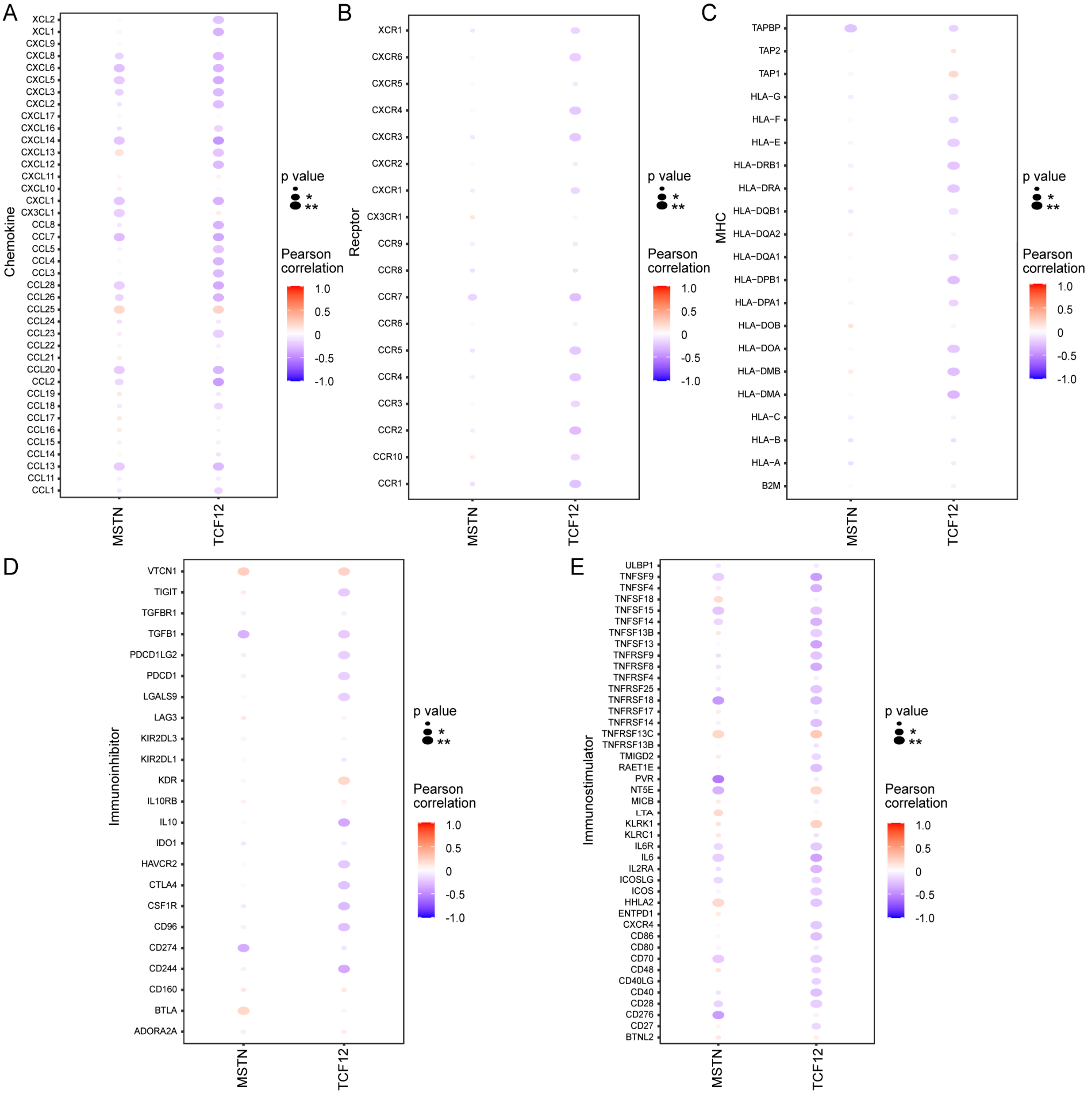

Figure S3. The relationship between MSTN and TCF12 with immune factors. (**A**–**E**) The correlation of MSTN and TCF12 with chemokines, immuno-inhibitors, immuno-stimulators, MHC molecules and receptors.

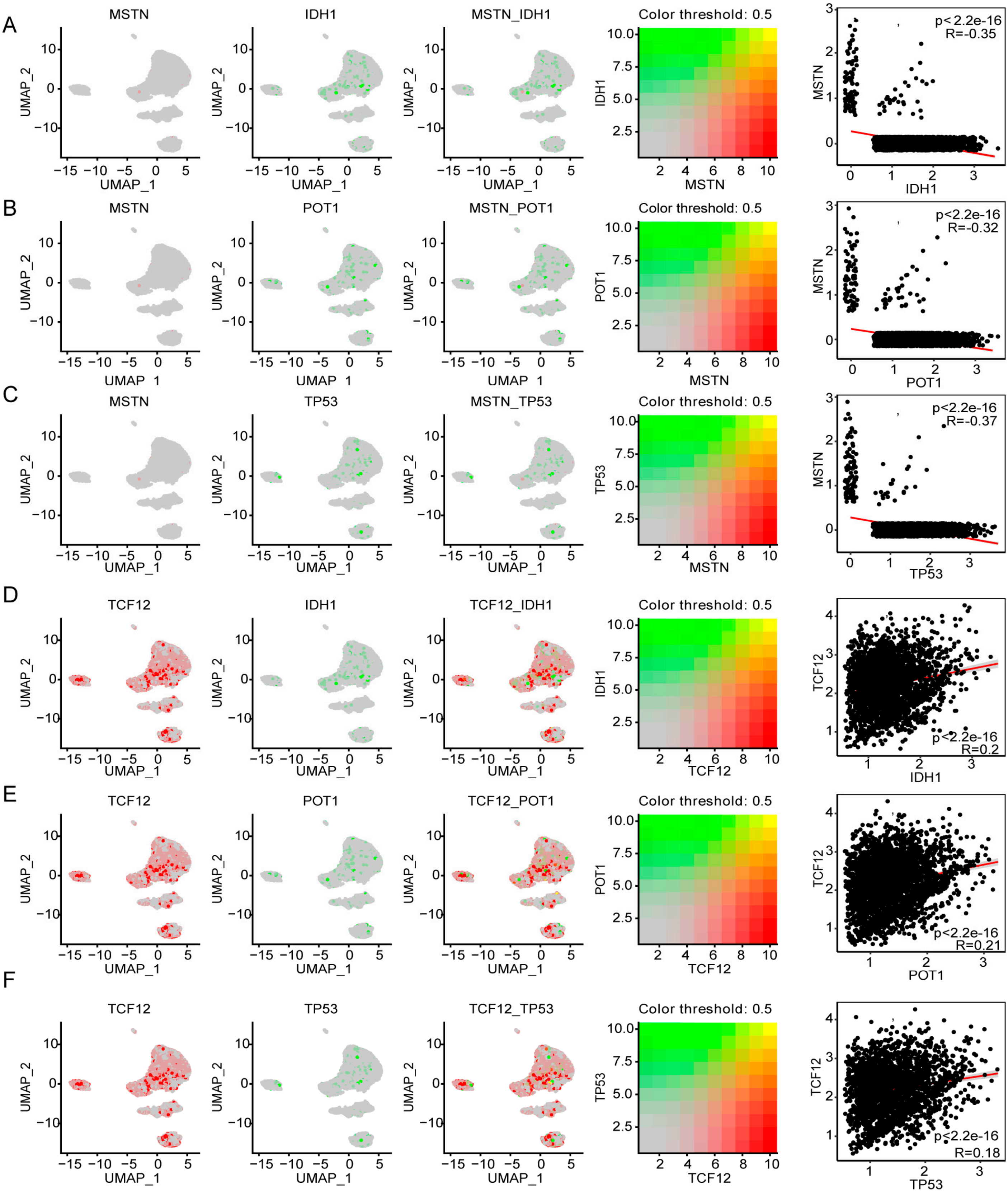

Figure S4. Co-expression analysis of MSTN and TCF12 with disease-related genes in single cells. (**A–C**) Co-expression of disease genes with the MSTN gene in single-cell data, as well as the correlation of co-expressed genes. (**D–F**) Co-expression of disease genes with the TCF12 gene in single-cell data, as well as the correlation of co-expressed genes.
