# Supplementary material for: MSTN and TCF12 as Candidate Immunometabolic Signatures in Glioma-Associated Foam Cells: Insights from Integrated Multi-Omics Analysis
Source: Curr Issues Mol Biol. 2026 Mar 9;48(3):289. doi: 10.3390/cimb48030289 (PMC13025612; doi:10.3390/cimb48030289)
Supplement: Supplementary file 1 [file cimb-48-00289-s001.zip › Supplementary Table S1. All the parameter settings and complete version information for all packages.pdf]

R	Version
RcisTarge1	1.22.0
RcisTarge1	1.19.2
limma	3.58.1
clusterPr(	4.10.1
ggplot2	3.5.0
ggrepel	0.9.5
ggraph	2.1.0
GSVA	1.50.0
corrplot	0.92
dplyr	1.1.4
patchwork	1.2.0
GSEABase	1.64.0
Seurat	4.3.0
SeuratObje	4.1.4
harmony	1.1.0
reshape2	1.4.4
scRNAtool\	0.0.7
parallel	4.3.2
KernSmoot	2.23-22
CellChat	1.6.1
monocle	2.30.0
pheatmap	1.0.12
cowplot	1.1.1
oncoPredi(	1.2
rms	6.7-1
Hmisc	5.1-1
timeROC	0.4
rmda	0.6
ggDCA	1.1
caret	6.0-94
survival	3.5-7
randomFore	3.3.0
randomSurv	3.6.4
tidyverse	2.0.0
survminer	0.4.9
spacexr	2.2.1
viridis	0.6.4
RColorBrew	1.1-3
