# Supplementary material for: MSTN and TCF12 as Candidate Immunometabolic Signatures in Glioma-Associated Foam Cells: Insights from Integrated Multi-Omics Analysis
Source: Curr Issues Mol Biol. 2026 Mar 9;48(3):289. doi: 10.3390/cimb48030289 (PMC13025612; doi:10.3390/cimb48030289)
Supplement: Supplementary file 1 [file cimb-48-00289-s001.zip › Supplementary Table S2. TAFC all marker genes.pdf]

Table S2. TAFC all marker genes					
p_val	avg_log2FC	pct.1	pct.2	p_val_adj	cluster
2.67E-35	0.899221	0.503	0.335	5.54E-31	Tumor associated foam cell
4.42E-36	0.6327954	0.924	0.983	9.14E-32	Tumor associated foam cell
3.05E-61	0.7237017	0.999	0.999	6.31E-57	Tumor associated foam cell
2.53E-80	0.613673	0.953	0.974	5.23E-76	Tumor associated foam cell
4.71E-91	0.6684093	0.583	0.196	9.76E-87	Tumor associated foam cell
6.88E-98	0.6369393	0.936	0.972	1.42E-93	Tumor associated foam cell
5.45E-102	0.5852737	0.577	0.191	1.13E-97	Tumor associated foam cell
7.04E-107	0.615077	0.614	0.208	1.46E-102	Tumor associated foam cell
6.22E-117	0.6769926	0.552	0.129	1.29E-112	Tumor associated foam cell
6.32E-120	0.6171279	0.959	0.959	1.31E-115	Tumor associated foam cell
4.58E-120	0.5997805	0.586	0.141	9.49E-116	Tumor associated foam cell
1.51E-120	0.5984421	0.516	0.084	3.13E-116	Tumor associated foam cell
2.91E-123	0.6053835	0.508	0.078	6.02E-119	Tumor associated foam cell
1.93E-126	0.5979337	0.53	0.082	4.00E-122	Tumor associated foam cell
1.32E-126	0.624879	0.52	0.078	2.73E-122	Tumor associated foam cell
1.73E-127	0.7939571	0.689	0.324	3.58E-123	Tumor associated foam cell
2.69E-132	0.5915234	0.96	0.96	5.56E-128	Tumor associated foam cell
2.30E-132	0.6131999	0.955	0.953	4.75E-128	Tumor associated foam cell
3.33E-133	0.6295469	0.572	0.107	6.90E-129	Tumor associated foam cell
8.75E-134	0.6224417	0.995	0.991	1.81E-129	Tumor associated foam cell
2.17E-136	0.6456143	0.563	0.096	4.49E-132	Tumor associated foam cell
7.57E-138	0.6678406	0.518	0.058	1.57E-133	Tumor associated foam cell
5.73E-138	0.6927658	0.581	0.112	1.19E-133	Tumor associated foam cell
5.42E-138	0.7457506	0.611	0.151	1.12E-133	Tumor associated foam cell
1.74E-139	0.6286395	0.933	0.918	3.61E-135	Tumor associated foam cell
1.66E-139	0.7843671	0.938	0.963	3.44E-135	Tumor associated foam cell
1.01E-143	0.6295281	0.957	0.941	2.09E-139	Tumor associated foam cell
5.11E-145	0.5919621	0.978	0.978	1.06E-140	Tumor associated foam cell
2.82E-147	0.7211591	0.952	0.964	5.83E-143	Tumor associated foam cell
6.21E-149	0.6944765	0.562	0.077	1.29E-144	Tumor associated foam cell
5.70E-152	0.7786296	1	1	1.18E-147	Tumor associated foam cell
1.19E-155	0.6669386	0.926	0.916	2.46E-151	Tumor associated foam cell
2.22E-159	0.6905699	0.975	0.974	4.59E-155	Tumor associated foam cell
3.14E-162	0.6200068	0.981	0.975	6.49E-158	Tumor associated foam cell
4.82E-165	0.6863058	0.942	0.935	9.99E-161	Tumor associated foam cell
3.45E-165	0.6130235	0.992	0.992	7.14E-161	Tumor associated foam cell
1.05E-166	0.6745154	0.961	0.946	2.17E-162	Tumor associated foam cell
6.04E-169	0.7547187	0.976	0.965	1.25E-164	Tumor associated foam cell
2.72E-169	0.6411394	0.996	0.998	5.63E-165	Tumor associated foam cell
8.29E-170	0.6652871	0.979	0.971	1.72E-165	Tumor associated foam cell
4.46E-172	0.6629088	0.976	0.971	9.23E-168	Tumor associated foam cell
1.53E-174	0.75664	0.958	0.95	3.16E-170	Tumor associated foam cell
3.85E-178	0.7256076	0.976	0.971	7.97E-174	Tumor associated foam cell
1.53E-179	0.6157559	0.997	0.999	3.17E-175	Tumor associated foam cell
7.30E-181	0.7295749	0.969	0.964	1.51E-176	Tumor associated foam cell
4.78E-181	0.7391419	0.991	0.992	9.89E-177	Tumor associated foam cell

1.29E-186	0.6501847	0.999	0.999	2.67E-182	Tumor associated foam cell
8.49E-188	0.634191	0.997	0.989	1.76E-183	Tumor associated foam cell
5.54E-188	1.0982063	0.996	0.994	1.15E-183	Tumor associated foam cell
7.87E-197	0.8003388	0.999	0.994	1.63E-192	Tumor associated foam cell
1.53E-198	0.6481423	1	0.999	3.18E-194	Tumor associated foam cell
1.89E-199	0.8156415	1	0.994	3.91E-195	Tumor associated foam cell
2.16E-201	0.6851236	1	1	4.47E-197	Tumor associated foam cell
8.68E-202	0.6172567	1	1	1.80E-197	Tumor associated foam cell
8.27E-204	0.647749	1	0.999	1.71E-199	Tumor associated foam cell
8.26E-204	0.69325	0.995	0.99	1.71E-199	Tumor associated foam cell
2.16E-205	0.7229672	1	1	4.46E-201	Tumor associated foam cell
3.62E-212	0.6390827	0.999	0.998	7.49E-208	Tumor associated foam cell
7.06E-214	0.8591857	0.98	0.968	1.46E-209	Tumor associated foam cell
9.81E-218	0.7239761	0.997	0.994	2.03E-213	Tumor associated foam cell
3.88E-218	0.6953363	0.997	0.995	8.04E-214	Tumor associated foam cell
3.20E-218	0.7795406	0.995	0.993	6.62E-214	Tumor associated foam cell
8.40E-221	0.6102895	1	1	1.74E-216	Tumor associated foam cell
2.61E-224	0.6773455	1	1	5.41E-220	Tumor associated foam cell
8.52E-232	0.7356799	1	1	1.76E-227	Tumor associated foam cell
3.52E-232	0.6383005	1	1	7.29E-228	Tumor associated foam cell
2.34E-235	0.7429045	0.999	0.998	4.84E-231	Tumor associated foam cell
1.35E-236	0.73975	1	0.996	2.80E-232	Tumor associated foam cell
2.49E-242	0.7028721	1	0.994	5.15E-238	Tumor associated foam cell
9.23E-244	0.6699954	1	0.999	1.91E-239	Tumor associated foam cell
1.58E-245	0.7287841	1	0.999	3.27E-241	Tumor associated foam cell
4.07E-248	0.8335974	1	1	8.43E-244	Tumor associated foam cell
1.25E-249	0.7001941	1	0.999	2.59E-245	Tumor associated foam cell
1.81E-250	0.7559375	1	1	3.75E-246	Tumor associated foam cell
8.84E-253	0.6970193	1	0.999	1.83E-248	Tumor associated foam cell
4.01E-255	0.7204513	1	1	8.31E-251	Tumor associated foam cell
2.04E-255	0.7989056	1	1	4.23E-251	Tumor associated foam cell
1.77E-255	0.6871543	1	1	3.67E-251	Tumor associated foam cell
1.97E-259	0.7885853	1	1	4.08E-255	Tumor associated foam cell
9.02E-261	0.7939639	1	0.998	1.87E-256	Tumor associated foam cell
3.57E-263	0.7461091	1	0.998	7.39E-259	Tumor associated foam cell
5.15E-264	0.7156257	1	1	1.07E-259	Tumor associated foam cell
2.50E-271	0.9330238	0.991	0.985	5.18E-267	Tumor associated foam cell
1.24E-276	0.9521547	1	0.998	2.56E-272	Tumor associated foam cell
7.71E-279	0.7927683	1	0.998	1.60E-274	Tumor associated foam cell
5.65E-279	0.7413859	1	0.999	1.17E-274	Tumor associated foam cell
2.03E-286	1.1452418	0.994	0.995	4.21E-282	Tumor associated foam cell
1.71E-291	0.8639927	1	0.996	3.54E-287	Tumor associated foam cell
1.59E-293	0.8407499	1	0.999	3.30E-289	Tumor associated foam cell
9.97E-298	0.7988061	1	0.998	2.06E-293	Tumor associated foam cell
9.86E-301	0.7976333	1	0.999	2.04E-296	Tumor associated foam cell
1.81E-305	0.8648087	1	0.998	3.76E-301	Tumor associated foam cell
1.09E-306	0.9183114	1	0.999	2.25E-302	Tumor associated foam cell
6.64E-307	1.0306389	1	0.997	1.37E-302	Tumor associated foam cell

0	1.6983238	1	1	0	Tumor associated foam cell
0	1.3306371	1	0.998	0	Tumor associated foam cell
0	1.1728572	1	0.999	0	Tumor associated foam cell
0	1.1672425	1	0.999	0	Tumor associated foam cell
0	1.1648318	1	1	0	Tumor associated foam cell
0	1.1544881	1	0.999	0	Tumor associated foam cell
0	1.1427415	1	1	0	Tumor associated foam cell
0	1.1064028	1	1	0	Tumor associated foam cell
0	1.083922	1	0.999	0	Tumor associated foam cell
0	1.0684017	1	1	0	Tumor associated foam cell
0	1.0587522	1	0.999	0	Tumor associated foam cell
0	1.024133	1	0.999	0	Tumor associated foam cell
0	1.022663	1	1	0	Tumor associated foam cell
0	1.0217113	1	0.997	0	Tumor associated foam cell
0	1.020993	1	0.999	0	Tumor associated foam cell
0	1.0045801	1	0.999	0	Tumor associated foam cell
0	1.0032234	1	1	0	Tumor associated foam cell
0	0.9773557	1	0.999	0	Tumor associated foam cell
0	0.9711573	1	0.998	0	Tumor associated foam cell
0	0.9700565	1	1	0	Tumor associated foam cell
0	0.9575202	1	1	0	Tumor associated foam cell
0	0.9498474	1	1	0	Tumor associated foam cell
0	0.9222459	1	0.998	0	Tumor associated foam cell
0	0.9177604	1	0.997	0	Tumor associated foam cell
0	0.908351	1	1	0	Tumor associated foam cell
0	0.8782681	1	1	0	Tumor associated foam cell
0	0.868646	1	0.999	0	Tumor associated foam cell
0	0.8667675	1	0.998	0	Tumor associated foam cell
0	0.8622361	1	0.999	0	Tumor associated foam cell

gene
IGFBP2
LDHA
MT2A
C1QL1
APOC1
HMGN2
UBE2C
TYMS
RRM2
SEC61G
CHD7
CXXC4
SMC4
TMEM100
LINC00511
AC104051.2
CNBP
TCF12
DDX39A
PTN
TOX3
MSTN
PTTG1
TOP2A
MFF
DLL3
SOX2
BTF3
HES6
CDKN2C
MT-CO2
NME4
POLR2F
SEM1
SNRPG
FXYD6
POLR2J
SNHG6
UQCRB
LSM7
PPP1R14B
SOX4
YBX1
NDUFB2
TMEM258
UQCRH

RPS24
SRI
MT1X
RPS20
RPS14
CRYAB
RPL31
RPL8
RPL7
LRIG1
RPS29
MARCKS
SOX8
NME2
H3F3A
H2AFZ
RPL36
RPL21
RPL39
RPL4
NACA
C1orf61
RACK1
RPL3
RPL6
TPT1
RPL35
RPS21
RPLP1
RPL19
RPL37
RPL15
RPS6
RPS3
RPL35A
RPL41
COMMD6
RPL26
RPSA
RPLP0
BCAN
RPL14
RPL5
RPL9
FAU
RPL24
RPS4X
RPS18

EEF1A1
RPS15A
RPL11
RPL34
RPS19
RPL36A
RPL37A
RPS8
RPL32
RPS3A
RPS23
RPS25
RPS15
RPS13
RPL29
RPL10
RPL7A
RPS27A
RPL17
RPL30
RPS2
RPL22
RPS16
RPS7
RPLP2
RPL13
DBI
RPL18A
RPS28
