# Supplementary material for: MSTN and TCF12 as Candidate Immunometabolic Signatures in Glioma-Associated Foam Cells: Insights from Integrated Multi-Omics Analysis
Source: Curr Issues Mol Biol. 2026 Mar 9;48(3):289. doi: 10.3390/cimb48030289 (PMC13025612; doi:10.3390/cimb48030289)
Supplement: Supplementary file 1 [file cimb-48-00289-s001.zip › Supplementary Table S3. The binding motif of MSTN and TCF12 transcription factors.pdf]

Table S3. The binding motif of MSTN and TCF12 tra

| Number | logo | motif | NES | AUC |
|---|---|---|---|---|
| 1 | 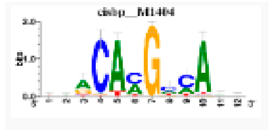 | cisbp_M1404 | 5.82 | 0.896 |
| 2 | 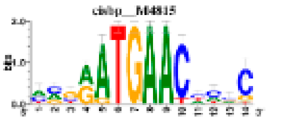 | cisbp_M4815 | 5.73 | 0.883 |
| 3 | 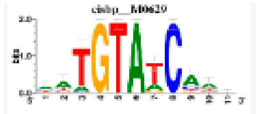 | cisbp_M0629 | 5.39 | 0.835 |
| 4 | 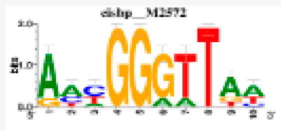 | cisbp_M2572 | 5.2 | 0.807 |
| 5 | 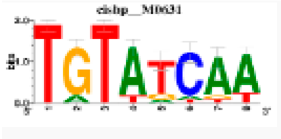 | cisbp_M0631 | 5.19 | 0.806 |
| 6 | 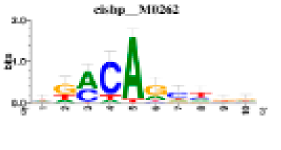 | cisbp_M0262 | 5.18 | 0.805 |
| 7 | 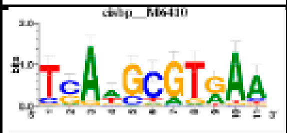 | cisbp_M6410 | 5.04 | 0.785 |
| 8 | 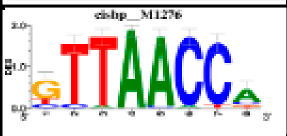 | cisbp_M1276 | 4.94 | 0.77 |
| 9 | 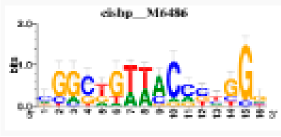 | cisbp_M6486 | 4.91 | 0.767 |
| 10 | 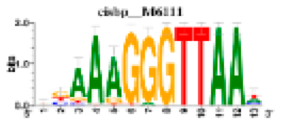 | cisbp_M6111 | 4.9 | 0.764 |
| 11 | 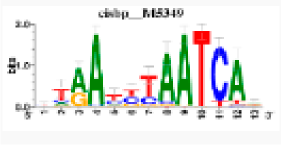 | cisbp_M5349 | 4.85 | 0.757 |
| 12 | 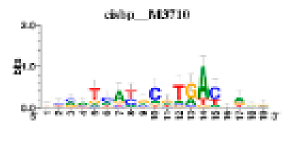 | cisbp_M3710 | 4.77 | 0.746 |

| | | | | |
|---|---|---|---|---|
| 13 | 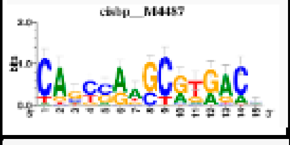 | cisbp_M4487 | 4.76 | 0.744 |
| 14 | 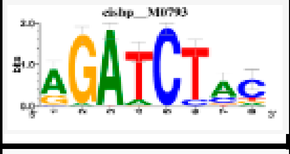 | cisbp_M0793 | 4.74 | 0.741 |
| 15 | 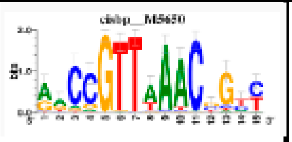 | cisbp_M5650 | 4.67 | 0.731 |
| 16 | 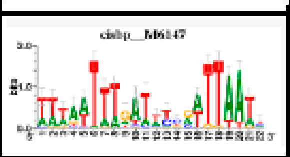 | cisbp_M6147 | 4.65 | 0.728 |
| 17 | 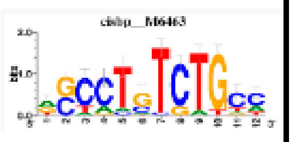 | cisbp_M6463 | 4.63 | 0.726 |
| 18 | 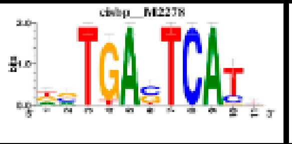 | cisbp_M2278 | 4.48 | 0.704 |
| 19 | 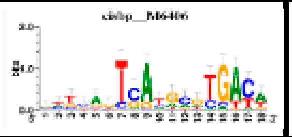 | cisbp_M6406 | 4.36 | 0.687 |
| 20 | 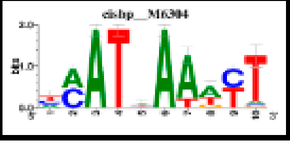 | cisbp_M6304 | 4.31 | 0.68 |

nscription factors

| TF_highConf |
|---|
| |
| |
| DMRT3(direcAnnotation) |
| ZNF554（inferredBy_Orthology） |
| DMRT1(direcAnnotation) |
| |
| PAX6(direcAnnotation) |
| |
| SPZ1(direcAnnotation) |
| ZNF652（inferredBy_Orthology） |
| |
| PAX2(direcAnnotation) |

| |
|---|
| PAX5(direcAnnotation) |
| |
| MYBL2(direcAnnotation) |
| ARID3A(direcAnnotation) |
| SMAD1(direcAnnotation) |
| FOS(direcAnnotation) |
| PAX2(direcAnnotation) |
| HOXD9(direcAnnotation) |
